# Global Identification of Prokaryotic Glycoproteins Based on an *Escherichia coli* Proteome Microarray

**DOI:** 10.1371/journal.pone.0049080

**Published:** 2012-11-07

**Authors:** Zong-xiu Wang, Rui-ping Deng, He-Wei Jiang, Shu-Juan Guo, Huang-ying Le, Xiao-dong Zhao, Chien-Sheng Chen, Ji-bin Zhang, Sheng-ce Tao

**Affiliations:** 1 State Key Laboratory of Agricultural Microbiology, College of Life Science and Technology, Huazhong Agricultural University, Wuhan, China; 2 Shanghai Center for Systems Biomedicine, Key Laboratory of Systems Biomedicine (Ministry of Education), Shanghai Jiao Tong University, Shanghai, China; 3 State Key Laboratory of Oncogenes and Related Genes, Shanghai Jiao Tong University, Shanghai, China; 4 Graduate Institute of Systems Biology and Bioinformatics, National Central University, Jhongli City, Taoyuan Country, Taiwan; Indian Institute of Science, India

## Abstract

Glycosylation is one of the most abundant protein posttranslational modifications. Protein glycosylation plays important roles not only in eukaryotes but also in prokaryotes. To further understand the roles of protein glycosylation in prokaryotes, we developed a lectin binding assay to screen glycoproteins on an *Escherichia coli* proteome microarray containing 4,256 affinity-purified *E.coli* proteins. Twenty-three *E.coli* proteins that bound Wheat-Germ Agglutinin (WGA) were identified. PANTHER protein classification analysis showed that these glycoprotein candidates were highly enriched in metabolic process and catalytic activity classes. One sub-network centered on deoxyribonuclease I (sbcB) was identified. Bioinformatics analysis suggests that prokaryotic protein glycosylation may play roles in nucleotide and nucleic acid metabolism. Fifteen of the 23 glycoprotein candidates were validated by lectin (WGA) staining, thereby increasing the number of validated *E. coli* glycoproteins from 3 to 18. By cataloguing glycoproteins in *E.coli*, our study greatly extends our understanding of protein glycosylation in prokaryotes.

## Introduction

Glycosylation is one of the most abundant protein post-translational modifications (PTMs) [1]. Among the various forms of protein glycosylation, *N*-linked glycosylation on Asn residues and *O*-linked glycosylation on Ser/Thr residues are the most common. It is predicted that more than two-thirds of eukaryotic proteins are glycosylated [2] and that these modifications are essential for a multitude of biological processes such as fertilization [3], cancer metastasis [4] and host-pathogen interactions [5], [6].

Protein glycosylation was first demonstrated in the late 1930s [7] and was long thought to occur only in eukaryotes [8]. The first identified prokaryotic glycoprotein was the cell surface protein of the archaeon *Halobacterium halobium* [9]. Protein glycosylation also occurs in bacteria, and more than 70 bacterial glycoproteins have been reported [10]. The vast majority of these glycoproteins are surface or secreted proteins and may play important roles in cell-cell interactions, surface adhesion, or evasion of immune responses [10]–[12].

Although many prokaryotic glycoproteins have been identified in recent years [13], [14], only three glycoporteins from the prokaryotic model organism *E.coli* are well recognized *i.e.* adhesin involved in diffuse adherence I (AIDA-I) [15], the TibA adhesion-invasion protein of enterotoxigenic *E. coli* [16], and the autoaggregation factor antigen 43 (Ag43) [17].

Functionally, these three *E.coli* glycoproteins are critical for host-pathogen interactions and biofilm formation. However, AIDA-I and TibA are only present in pathogenic *E.coli* strains, and Ag43 is the only known glycoprotein that exists in both pathogenic and non-pathogenic *E.coli* strains.

Several lines of evidence suggest that protein glycosylation may be a widespread phenomenon in prokaryotes [11], [18]. Protein glycosylation may be as abundant in prokaryotes as protein acetylation. Both protein glycosylation and acetylation were previously thought not to occur in prokaryotes, however, prokaryotic protein acetylation has recently been shown to be as abundant as that in eukaryotes [19], [20]. In spite of its importance, both the number and types of proteins that are glycosylated are very limited in prokaryotes. While the eukaryotic model organism, *Saccharomyces cerevisiae* has 350 validated glycoproteins [1], there is only one known glycoprotein in the non-pathogenic strain of the prokaryotic model organism *E.coli*. It is thus likely that the full range of biochemical and cellular functions of protein glycosylation in prokaryotes is barely understood, and it is highly likely that there are many more prokaryotic glycoproteins to be discovered. Although it is too early to predict the full extent of prokaryotic glycosylation, it is clear from the diversity of prokaryotic glycoproteins discovered in recent years that glycosylation in these organisms is the norm rather than the exception.

Proteome microarrays, also known as protein chips, are constructed by spotting hundreds or thousands of individually purified proteins in high density on a solid surface [21]–[23]. Proteome microarrays have been employed to probe various types of protein biochemical properties and enable high-throughput screening of thousands of proteins in a single experiment [24], [25]. Proteome microarrays provide information about direct biochemical and physical interactions among biomolecules and have been successfully applied in a variety of studies related to post-translational modifications [26]–[28], and in the global identification of glycoproteins in *Saccharomyces cerevisiae* [1].

Here, we used an *E. coli* proteome microarray and lectin affinity probing to globally identify glycoproteins in *E. coli*. Using our strategy, we identified 23 glcyoproteins and fifteen of them were successfully validated, thus greatly expanded the known glycoproteins in *E.coli*. Bioinformatics analysis showed that protein glycosylation may play important roles in nucleic acid metabolism.

## Materials and Methods

### Chemicals and Reagents

All chemicals were purchased from Sigma-Aldrich unless otherwise stated. FAST™ slides were purchased from GE Healthcare (Waukesha, WI, USA). The SmartArrayer 48 microarrayer and Slider Washer™ 8 slide clean up station were purchased from CapitalBio Co. (Beijing, China). Calf histones H3 and H4 were purchased from Roche (Basel, Switzerland), the GenePix 4200A slide scanner and GenePix Pro 6.0 were obtained from Axon Instruments (Elk Grove Village, Illinois, USA), and anti-6xHis antibody was from Abmart Co. (Shanghai, China). IRDye® 800CW Donkey anti-mouse antibody and the Odyssey Infrared Imaging System were from LI-COR, (Lincoln, Nebraska, USA), and Cy3-WGA was from Invitrogen (Carlsbad, California, USA).

### Proteome Microarray Construction

*E.coli* proteome microarrays were prepared as previously described [22]. Briefly, 4,256 proteins representing the majority of the *E. coli* K-12 MG1655 proteome were affinity purified using 6xHis tags and Ni-NTA binding. After elution and quality control by Western blotting with an anti-6xHis antibody, each protein was spotted twice on a FAST™ slide using a SmartArrayer 48 microarrayer. Calf histones H3 and H4 at a concentration of 1 μg/μL were used as positive controls. The printed microarrays were stored at −80°C until required.

### Glycoprotein Identification using the *E.coli* Proteome Microarrays

Protein microarrays were blocked with TBST and 1% BSA for 1 h and incubated with lectin solution (1 μg/mL) in the presence or absence of glycan competitors, the competitor for WGA is chitin hydrolysate, a highly concentrated solution of N-acetylglucosamine (glcNAc) and glcNAc oligomers, for 1 h at room temperature. The protein microarrays were then subjected to three 5-min washes in TBST with gentle shaking, and dried by centrifugation. The slides were scanned with a GenePix 4200A fluorescent microarray scanner.

### Data Analysis

Data were extracted by GenePix Pro 6.0 from the microarray images. The signal to noise ratio (SNR) was defined as F635 Median/B635 Median and was calculated for all the spots on the *E. coli* proteome microarray. The SNR of a protein was averaged from the two duplicated spots and then calculated for the two microarrays with and without glycan competitors as SNR (+) and SNR (−), respectively. The Calling Score was set as SNR (−)/SNR (+) and was calculated for all the proteins. Glycoprotein candidates were identified by examining the SNR (−) and calling scores for each protein.

### Protein Classification

The 23 glycoprotein candidates identified (**Table 1**) were classified using the PANTHER (Protein ANalysis THrough Evolutionary Relationships) classification system [29] with default settings. Gene symbols were used as input for the classification system.

**Table 1:** Glycoproteins identified on *E.coli* proteome microarray by lectin probing.

NO.	ID	SNR(−)	SNR(+)	Calling score>2.5	Annotation
1	EentC	152.91	36.08	4.24	isochorismate synthase 1
2	YgdK	147.60	56.78	2.60	predicted Fe-S metabolism protein
3	RibD	117.46	42.31	2.78	5-amino-6-(5-phosphoribosylamino)uracil reductase
4	YbiJ	109.97	34.14	3.22	predicted protein
5	Exo	101.99	23.46	4.35	Uncharacterized exonuclease xni
6	DeoA	98.69	19.72	5.01	Thymidine phosphorylase
7	MaoC	87.76	29.99	2.93	fused aldehyde dehydrogenase/enoyl-CoA hydratase
8	SbcB	86.40	30.07	2.87	Exodeoxyribonuclease I
9	HolC	75.41	28.14	2.68	DNA polymerase III subunit chi
10	DnaC	70.21	23.74	2.96	DNA replication protein dnaC
11	YcaK	68.36	7.63	8.96	conserved protein
12	YbaJ	67.09	19.20	3.49	predicted protein
13	YkgD	62.64	10.62	5.90	predicted DNA-binding transcriptional regulator
14	Edd	62.55	8.40	7.45	6-phosphogluconate dehydratase
15	FimH	49.48	17.65	2.80	minor component of type 1 fimbriae
16	AroK	47.97	8.19	5.86	shikimate kinase I
17	TalB	47.56	3.94	12.08	transaldolase B
18	WzxC	39.35	15.32	2.57	colanic acid exporter
19	SurA	32.55	8.06	4.04	peptidyl-prolyl cis-trans isomerase (PPIase); Chaperone surA
20	YifC	33.63	12.86	2.62	Lipopolysaccharide biosynthesis protein
21	YbdK	25.22	6.95	3.63	Carboxylate-amine ligase ybdK
22	HisP	28.36	10.06	2.82	Histidine transport ATP-binding protein
23	YdiD	20.33	2.01	10.11	Short chain fatty acid CoA ligase

### Network Analysis

Protein interaction networks for the 23 glycoprotein candidates (**Table 1**) were generated by STRING [30]. Common downstream targets or upstream regulators of multiple proteins were identified using this software which facilitated the process of selecting proteins which are key factors or are involved in potential mechanisms from the large number of proteins. The analysis was performed with a confidence parameter of 0.15.

### Prediction of Secreted Proteins

Signal peptides for secreted proteins were predicted using SignalP [31]. Non-classical secreted proteins were predicted using SecretomeP [32]. Unless otherwise stated, all predictions using these programs were performed using default settings.

### Western Blotting

Proteins were separated on 10% and 15% SDS-PAGE gel, and transferred onto a PVDF transfer membrane after electrophoresis. After blocking with 5% non-fat milk at room temperature for 1 h, the membranes were incubated for 2 h at RT with a mouse monoclonal anti-6xHis antibody (1∶5000). Three 5-min washes with gentle shaking in TBST were carried out to remove excess primary antibody. The membrane was then incubated with a fluorescent labeled secondary antibody (IRDye® 800 labeled donkey anti-mouse, 1∶1000) for 2 h at RT. The signal was visualized and recorded with an Odyssey Infrared Imaging System.

### Lectin Blotting

Proteins were transferred to PVDF membranes. The membrane was blocked by PBS (pH 7.5) with 1% Tween-20 for 1 h at room temperature followed by incubation with 1 μg/μL HRP-conjugated lectin at room temperature for 2 h. After four 10-min washes with 0.05% Tween-20 in PBS (pH 7.5), the membrane was incubated with SuperSignal West Pico Chemiluminescent Substrate and exposed to an X-ray film.

## Results

### Proteome Microarray Strategy

**Figure 1:**
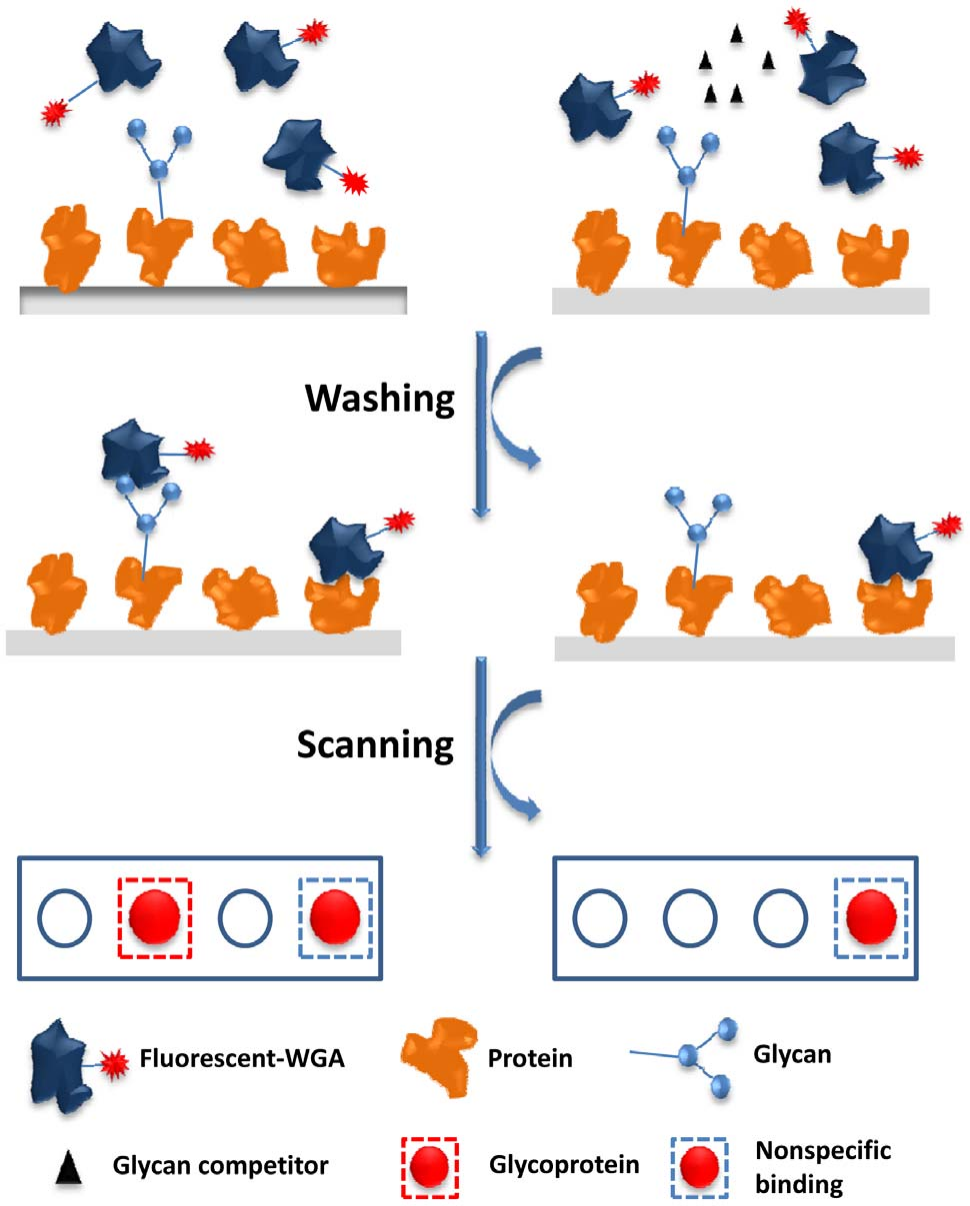
Schematic diagram of glycan competition assays. Proteome arrays were probed with fluorescent dye conjugated lectin. **Left track**; One proteome chip was incubated with lectins, then washed to remove free lectins and some weak, non-specific interactions. Stronger non-specific interactions still occurred. **Right track**; A second proteome chip was incubated with lectins in the presence of excess amounts of glycan competitors to block glycan-dependent interactions. Glycoproteins were readily identified by comparing the signal intensities between the two microarrays, without and with glycan competitors.

A schematic diagram of the experimental procedure followed is shown in **Fig. 1** **.** Briefly, two proteome microarrays were probed side by side with fluorescent conjugated lectin. A specific glycan competitor was included during the lectin probing of one of the microarrays as a control. After incubation, unbound lectin was removed by washing and the microarrays were scanned with a microarray scanner. Glycoproteins were identified by comparing the two microarrays with and without the addition of a glycan competitor.

### Twenty-three Glycoprotein Candidates were Identified by Probing the *E.coli* Proteome Microarray with a Fluorescent Labeled Lectin

A proteome microarray based strategy (**Fig. 1**) was applied to globally identify glycoproteins in *E. coli*. A *E. coli* proteome microarray with 4,256 affinity purified N-terminal 6xHis tagged *E.coli* proteins [22] was used in this study. Since all proteins were purified from their original *E. coli* host, we reasoned that the glycosylation status of the proteins should be similar to that under natural conditions, facilitating the global identification of novel glycoproteins in *E. coli*. One of the most commonly used lectins, WGA, which binds specifically to *O*-linked glycosylated proteins was selected for microarray probing. Cy5-conjugated WGA was incubated with the *E. coli* proteome microarray at an appropriate concentration. Since lectins are also proteins, it is possible that lectins may also nonspecifically bind to some proteins in the microarray, thus causing false positive results. To rule out this possibility, a control experiment was carried out in which the chitin hydrolysate (a highly concentrated solution of N-acetylglucosamine (glcNAc) and glcNAc oligomers) of WGA was added.

**Figure 2:**
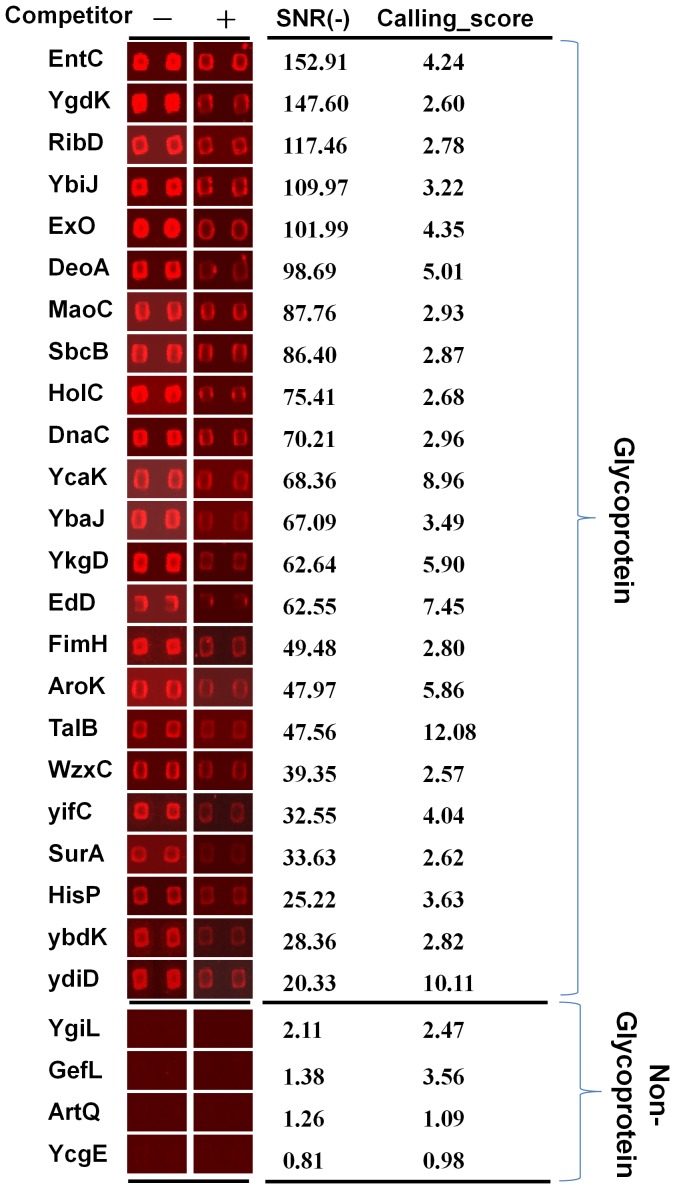
Glycoproteins identified by *E. coli* proteome microarrays and on-chip lectin (WGA) competition assays. Four representative novel glycoprotein candidates are shown. SNRs in the absence of glycan competitors and fold changes are given. The glycan competitor used for WGA probing was chitin hydrolysate.

Slides were extensively washed to minimize non-specific binding. The signal to noise ratio (SNR) for each spot was defined as the median foreground/median background, and the SNR of a protein was averaged from two duplicated spots. The SNR of the experiment without the glycan competitor and the SNR of the experiment with the glycan competitor were designated SNR (−) and SNR (+), respectively. Another index Calling Score was defined as SNR (−)/SNR (+) and was calculated for each protein. Twenty-three proteins with an SNR (−) ≥3 and a Calling Score ≥2.5 were identified as novel glycoproteins in WGA probing experiments (**Table 1**). The microarray images of the 23 glycoprotein candidates and four non-glycoproteins are shown in **Fig. 2** along with their SNR(−) values and Calling_Scores.

### Protein Classification Revealed that the Novel Glycoproteins are Highly Enriched in Metabolic Process and Catalytic Activity Classes

**Figure 3:**
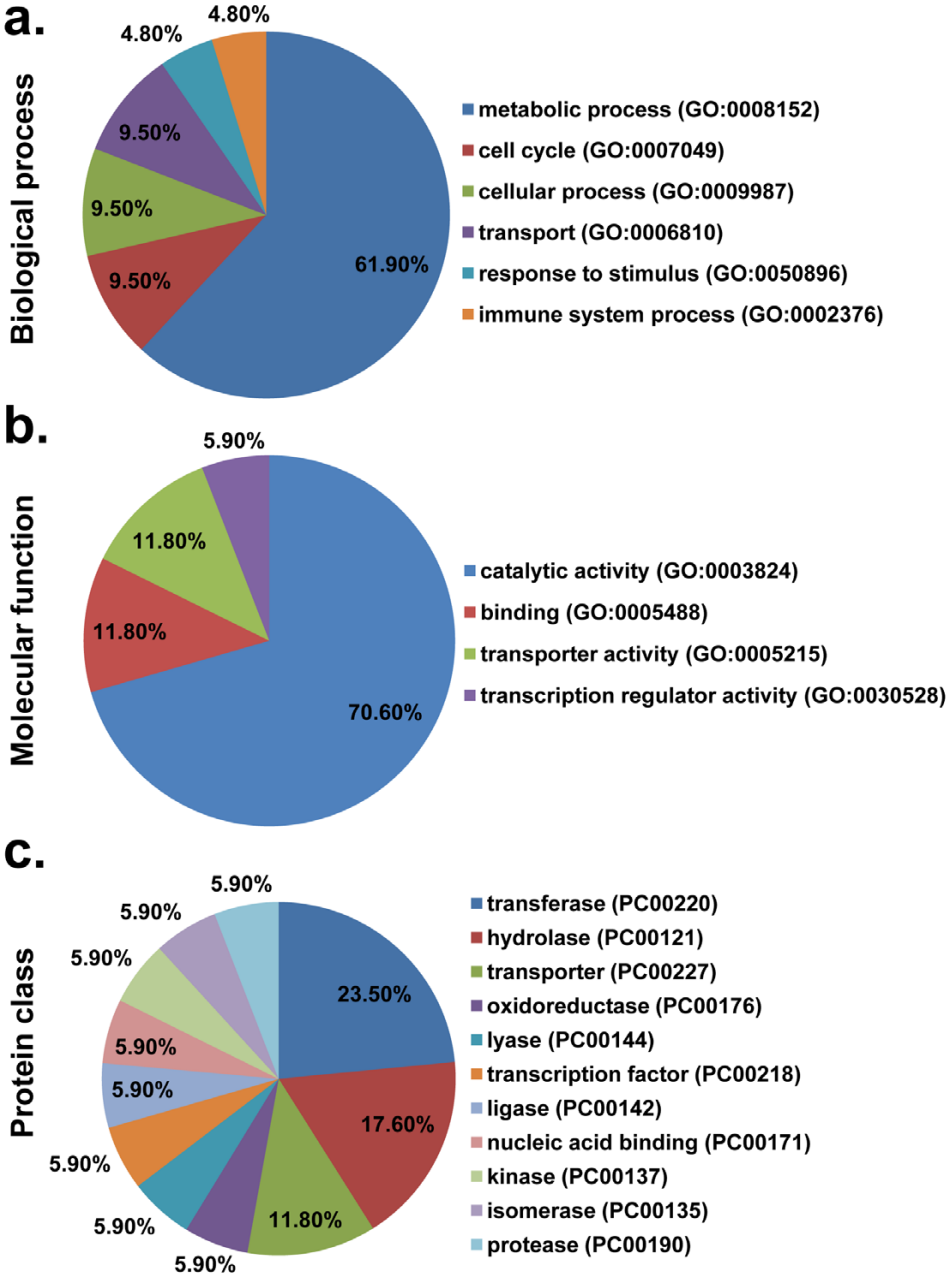
Functional distribution of candidate proteins according to their (a) Biological process, (b) Molecular function and (c) Protein class. Categorizations were based on information provided by the online resource PANTHER classification system.

To understand the biological relevance of these novel glycoproteins (**Table 1**), we used the PANTHER system [29], [33] to classify them according to the gene ontology and protein categories in which they are involved. The glycoprotein candidates were classified into six biological process groups (**Fig. 3A**). The largest group was metabolic processes (GO: 0008152) (61.9%). Significant numbers of these glycoproteins are also involved in the cell cycle (GO: 0007049) (9.5%), in cellular processes (GO: 0009987) (9.5%) and in transport (GO: 0006810) (9.5%). Further analysis revealed that the nucleobase, nucleoside, nucleotide and nucleic acid metabolic process category (GO: 0006139) is the largest GO daughter term within the metabolic process category (33.3%) (**Table S1**). The candidates could be classified into 4 molecular function groups (**Fig. 3B**). The largest group was catalytic activity (GO: 0003824) (70.6%), followed by binding (GO: 0005488) (11.8%) and transporter activity (GO: 0005212) (11.8%). Further analysis revealed that transferase activity (GO: 0016740) was one of the most abundant GO daughter terms within the molecular function category (30.77%) (**Table S2**). The candidates were classified into 11 PANTHER protein class groups (**Fig. 3C**), the top three of which were transferases (PC00220) (23.5%), hydrolases (PC00121) (17.6%) and transporters (PC00227) (11.8%). These data indicate that protein glycosylation in *E. coli* may play important roles and be involved in a wide range of biological functions.

### Protein Localization Prediction Suggests that Most of the 23 Glycoproteins may be Secreted Proteins

We also performed cellular component analysis on the 23 novel glycoproteins using PANTHER; however this analysis failed due to the lack of experimentally-validated protein localization information for *E. coli*. Since most of the known prokaryotic glycoproteins are either membrane-bound or secreted proteins, we reasoned that the 23 glycoprotein candidates may also be enriched in these two protein categories. We performed a variety of localization-related predictions to test this. SignalP 4.0 [34], [35] predicted that 10 of the 23 glycoproteins, *i.e.* YgdK, YbiJ, DeoA, HolC, YbaJ, Edd, TalB, YifC, YbdK and HisP, have signal peptides. Using Secretome 2.0 [36] to predict non-classically secreted proteins with a default determining score of 0.5, all 23 glycoprotein candidates were predicted to be secreted proteins.

### Network Analysis Highlighted a Subnetwork Centered on Nucleic Acid Metabolism

**Figure 4:**
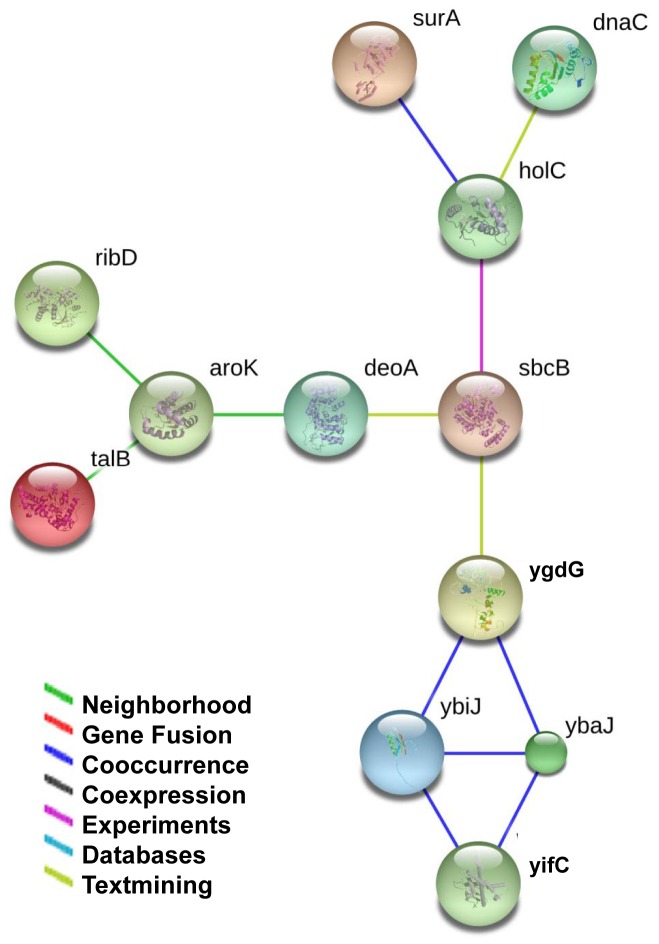
The protein-protein interaction network of the 23 glycoprotein candidates. The network was mapped using STRING, and the confidence parameter was set as 0.15. Lines between proteins stand for possible interactions, and are color-coded based on the type of interaction.

We constructed biological interaction networks for the glycoprotein candidates to create significance out of the protein list (**Fig. 4**). Candidate proteins listed in **Table 1** were imported into the online network analysis tool STRING [30] to build up the network. Candidates that could be networked were linked by various relationships such as neighborhood, gene fusion and co-expression relationships. After removing isolated nodes, one network was built up centered on deoxyribonuclease I (SbcB). Other important components included thymidine phosphorylase (DeoA), HolC (DNA polymerase III subunit Chi) and a protein which binds to exonuclease III and Single strand binding protein (Exo). Together with data from the biological process analysis in the PANTHER protein classification, the network analysis suggests novel functions of protein glycosylation in *E. coli* nucleic acid metabolism.

### Glycoprotein Candidates were Validated as Glycoproteins using Lectin Blotting

**Figure 5:**
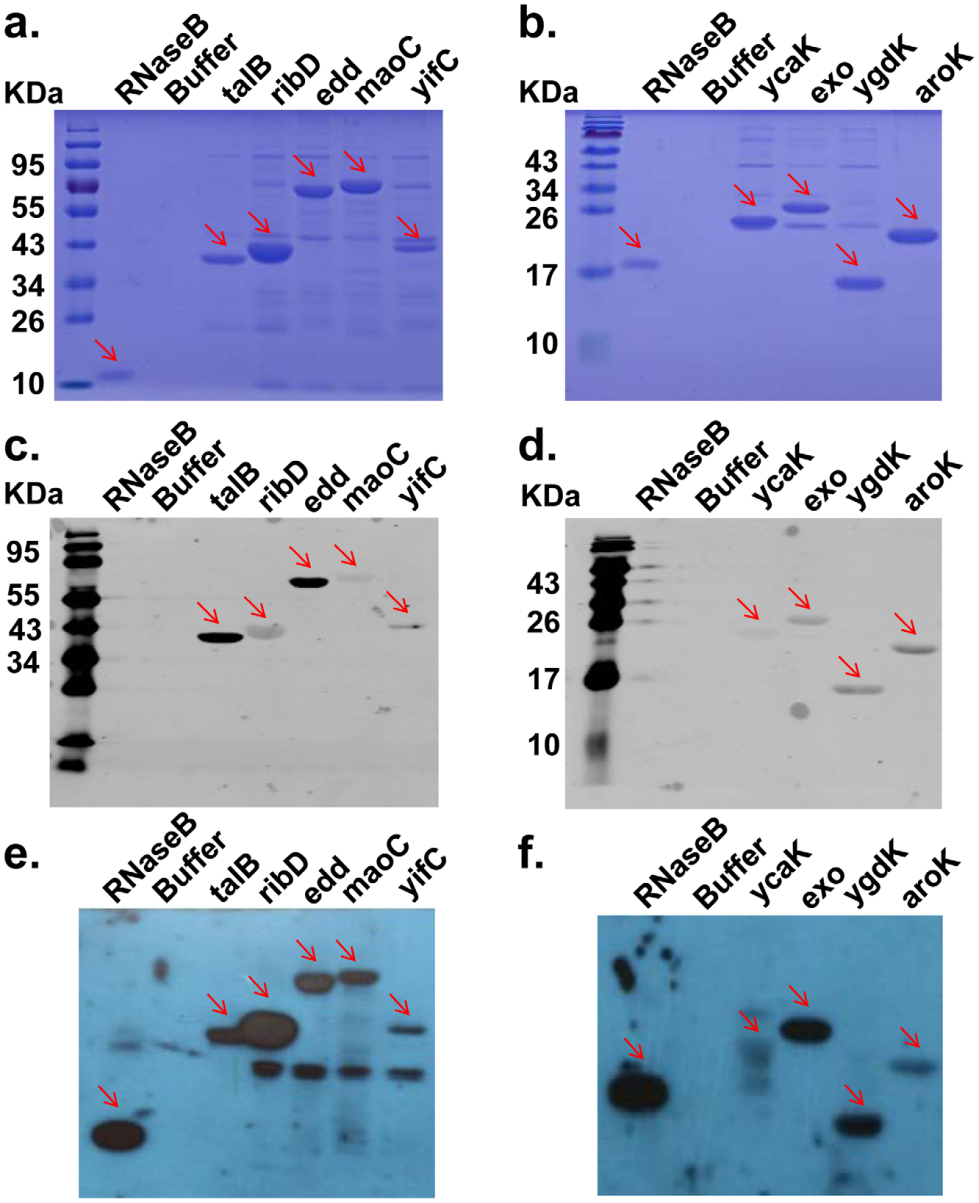
Validation of the novel glycoproteins by lectin blotting. A heavily glycosylated protein RNaseB was included as a positive control. The protein elution buffer used to purify the glycoproteins was used as a blank control. Red arrows indicate the target protein bands on the gel or the membrane. Results for the nine validated glycoproteins are shown. (**a–b**) Coomassie staining. (**c–d**) Western blotting using an anti-6xHis antibody. All of the glycoprotein candidates were 6xHis tagged at their C-terminals. (**e–f**) Lectin blotting using a biotinylated WGA followed by HRP-conjugated streptavidin.

To validate the glycoprotein candidates, the 23 proteins listed in **Table 1** were affinity-purified using N-terminal 6xHis tags, 15 of them were successfully purified. The glycosylation status of these candidate proteins was detected with a HRP-conjugated WGA. The heavily glycosylated protein RNaseB was included as a positive control. The purity of the nine validated proteins was confirmed by Coomassie staining (**Fig. 5a–b**), and their identity was confirmed by Western blotting using an anti-6xHis antibody (**Fig. 5c–d**). WGA lectin staining gave positive signals for nine of the candidates (**Fig. 5e–f**), *i.e.* TalB, RibD, Edd, MaoC, YifC, YcaK, Exo, YgdK and AroK. The purity and glycosylation status of all the 15 successfully purified proteins was confirmed by Coomassie staining (**Fig. S1a, S1c**) and WGA lectin staining (**Fig. S1b, S1d**), respectively. All the proteins were 6xHis tagged, they were purified by using Ni-NTA agarose. This protein affinity purification system is good for lots of biological applications, however, co-purification of other proteins is very common by using this system as shown by numerous studies from other groups. The extra bands of lectin blotting could be the co-purified proteins that happen to be glycosyalted and detected by WGA blotting.

## Discussion

Using a high-throughput *E. coli* proteome microarray and a glycan-specific lectin binding assay, we have globally screened for glycoproteins in *E. coli*. We identified 23 novel glycoproteins, fifteen of which were validated by lectin blotting. Together with the three known *E. coli* glycoproteins, a total of 18 glycoproteins have been validated in *E. coli*. Consistent with the fact that most of the known glycoproteins are either membrane bound or secreted proteins, around half of our glycoprotein candidates (10/23) were predicted to have signal peptides and all of them were predicted to be secreted proteins. Protein classification analysis identified metabolic processes and catalytic activity as the two most abundant GO terms. One sub-network centered on deoxyribonuclease I (sbcB) was identified, suggesting that prokaryotic protein glycosylation may play important roles in nucleotide and nucleic acid metabolism.

A combination of proteome microarrays and lectin probing is well suited for the global identification of *E. coli* glycoproteins for the following reasons: (1) *E.coli* proteins were purified from their original host, presumably preserving native glycosylations; (2) Each of the protein is physically addressable on the microarray, thus facilitating the identification of novel proteins; (3) the yield of the overexpressed proteins in *E.coli* is generally much higher than in other systems [37], [38], thus the local concentration of proteins on the *E. coli* proteome microarray is much higher than that of other proteome microarrays, *e.g.* yeast proteome microarrays [21] and human proteome microarrays [39]. This high local protein concentration greatly facilitates the identification of *E. coli* glycoproteins. Although some lectins may bind non-specifically to some proteins, the addition of glycan competitors can dramatically increase the fidelity of the binding results. Thus, *E. coli* glycoproteins are readily discovered by lectin probing.

Most known prokaryotic glycoproteins, such as the surface-layer (S-layer) proteins which are the best-studied bacterial glycoproteins [8], are either membrane-bound or secreted proteins. We expect that the majority of our newly identified *E. coli* glycoproteins are also membrane proteins or secreted proteins. Consistent with this expectation, most of the glycoprotein candidates were predicted to be non-classical secreted proteins (23/23) and to have signal peptides (10/23). Prediction of the location of glycoprotein candidates at least partially confirmed these findings. In addition, three candidate glycoproteins, *i.e*. WzxC (colanic acid exporter) [40], FimH (minor component of type 1 fimbriae) [41] and YifC (Lipopolysaccharide biosynthesis protein) [42] are known to be localized on the membrane of *E. coli* [43]. It is possible that membrane proteins are under-represent compared to other categories of proteins since they are difficult to purify successfully.

Among the three known *E. coli* glycoproteins, AIDA-I was originally identified as a plasmid-encoded protein, and confers a pattern of diffuse adherence on the surface of cultured epithelial cells [44]. This adhesion plays a role in diarrheal diseases in piglets, causing major economic losses in farms worldwide. Besides adhesin, AIDA-I has also been shown to mediate self-association, biofilm formation and invasion of epithelial cells. This protein undergoes an *O*-glycosylation by a specific cytoplasmic protein autotransporter adhesin heptosyltransferase (Aah) [15], [45]. Glycosylated TibA was originally identified in the classical ETEC enterotoxigenic *E. coli* serotype strain H10407 [46] and belongs to the autotransporter protein family. TibA is synthesized as a 100 kD precursor and seems to be an adhesin/invasin of human intestinal epithelial cells [47]. Ag43 is an autotransporter protein that facilitates cell-to-cell aggregation of *E. coli* [48]–[50]. Ag43 was identified as the product of the flu gene [49], [50]. It shows homology to several other members of the autotransporter protein family, such as the AIDA-I adhesin and the TibA adhesin of enterotoxigenic *E. coli.*

However, none of the three known *E. coli* glycoproteins were detected here. AIDA-I and TibA are only present in pathogenic *E. coli* strains. The *E. coli* proteome microarray was constructed using the non-pathogenic K12 MG1655 strain, and thus does not include AIDA-I and TibA. The structure and composition of the glycans carried by Ag43 are still not clear, thus it is possible that these glycans may not be recognized or may not be accessible to WGA. This may explain why Ag43 was not on our list of glycoprotein candidates (**Table 1**).

We initially probed the *E. coli* proteome microarray with the two most commonly used lectins, WGA and ConA, which bind specifically to *O*-linked glycosylations and *N*-linked glycosylations, respectively. However, none of the glycoprotein candidates identified by ConA probing could be validated successfully (data not shown). Oligosaccharyltransferase-mediated *O*-linked glycosylation is more widespread and abundant than *N*-linked glycosylation in Gram-negative bacteria such as *E. coli*, whilst the reverse is true for eukaryotes [51]. Thus it is not surprising that only WGA probing was successful. This also suggests that it may be possible to identify more glycoproteins on the *E. coli* proteome microarray using lectins other than WGA and ConA. Since our study was carried out at the systems level, *i.e.* the lectins were probed against almost all of the *E. coli* proteins (4,256 proteins), our data may serve as strong evidence in support of the observation that *O*-linked glycosylation is more common in prokaryotes than eukaryotes.

The 23 novel glycoproteins have diverse molecular functions and participate in a range of biological processes. It is thus likely that there may be more glycoproteins to discover in *E. coli* under different physiological circumstances. Of the glycoproteins we discovered, Exo, deoA, ExoI and DnaC are related to the metabolism of nucleic acids, and perform different but mutually relevant functions. TalB, RibD, Edd, MaoC, YcaK, YgdK, AroK, MaoC, EntC, SurA, YbdK, and FadK are members of different metabolic pathoways such as glycolysis, the pentose phosphate pathway, and fatty acid biosynthesis. Yifc is responsible for regulating the length of phosphoglyceride-linked Enterobacterial Common Antigen (ECAPG) polysaccharide chains. WzxC is an uncharacterized member of the Polysaccharide Transporter (PST) family and may be responsible for lipid polysaccharide LPS and protein glycosylation in bacteria. Both YifC and WzxC are involved in glycosylation processes. Even though the function of Ybij is unknown, its co-occurrence with Ybij, Ybaj and Yifc (**Fig. 4**) suggests that Ybij may also be related to polysaccharide transportation or biofilm formation. YkgD is a predicated transcriptional regulator. There is no experimental evidence to show that ykgD homolog from any other bacteria is also glycosylated. Interestingly, we have also identified several human transcriptional factors/regulators that could be glycosylated by a specific glycotransferase (unpublsished). YkgD is highly possible to be a secreted protein by bioinformatic predication, since most of the known prokaryotic glycoproteins are membrane protein or secreted proteins, thus it is not surprising that ykgD is also glycosylated. The glycosylation of ygkD may play a role of the regulation of its DNA binding activity, further functional study is ongoing to test this possibility. Global and unbiased screening has a much higher chance of obtaining unexpected findings as compared to traditional studies, this phenomenon has already been proved many times, the finding that an *E.coli* transcriptional regulator is glycosylated may be just another case. Both protein classification analysis and network analysis showed that the novel glycoproteins are highly enriched in nucleic acid and nucleoside metabolism classes, suggesting new roles for prokaryotic protein glycosylation which can be validated through future functional studies.

Glycoproteins synthesized by pathogenic *E. coli* strains are critical in host-pathogen interactions and pathogenicity. Pathogenic *E. coli* recognize and attach to the surfaces of intestinal tissues through specific glycoproteins [52], [53]. According to WHO, more than 2 million people, mainly infants, die of *E. coli*-associated diarrhea each year [54], [55]. It is highly likely that most of the glycoproteins identified from the non-pathogenic *E. coli* strain are also glycosylated in pathogenic strains. These glycoproteins may assist the major disease-causing glycoproteins that are only found in pathogenic strains.

Taken together, we have performed global identification of glycoproteins in *E. coli* using an *E. coli* proteome microarray and glycan-specific lectin binding. Our results greatly expand the number of validated *E. coli* glycoproteins, and suggest new roles for prokaryotic protein glycosylation. We believe that the novel *E. coli* glycoproteins identified in this study will serve as a starting point for a more comprehensive exploration of the role of protein glycosylation in prokaryotes. We foresee that the output of functional studies will benefit both basic research and clinically-related studies.

## Supporting Information

Figure S1 **Validation of the 15 successfully purified glycoprotein candidates by lectin blotting.** A heavily glycosylated protein RNaseB was included as a positive control. The protein elution buffer used to purify the glycoproteins was used as a blank control. Red arrows indicate the target protein bands on the gel or the membrane. (**a, c**) Coomassie staining. (**b, d**) Lectin blotting using a biotinylated WGA followed by HRP-conjugated streptavidin.
(PDF)

Table S1 **Biological process: primary metabolic process.**
(PDF)

Table S2 **Molecular function: catalytic activity.**
(PDF)
