# Supplementary figures and images for: Global Identification of Prokaryotic Glycoproteins Based on an Escherichia coli Proteome Microarray
Source: PLoS One. 2012 Nov 7;7(11):e49080. doi: 10.1371/journal.pone.0049080 (PMC3492326; doi:10.1371/journal.pone.0049080)

**a.**

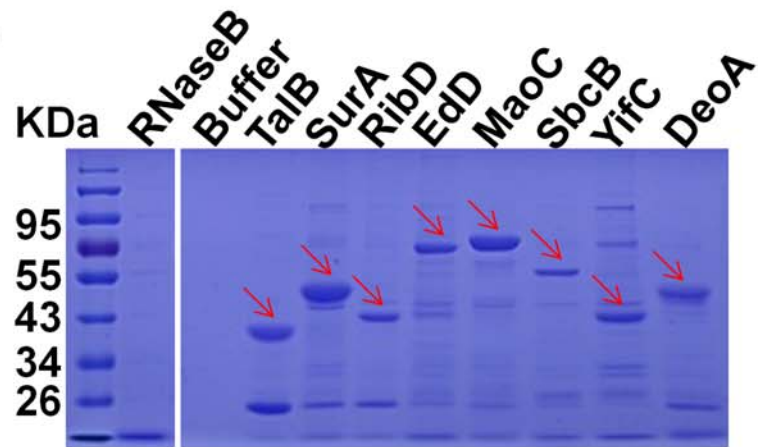

**b.**

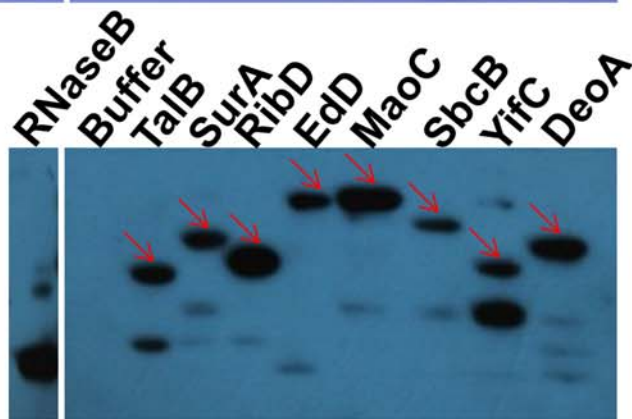

**c.**

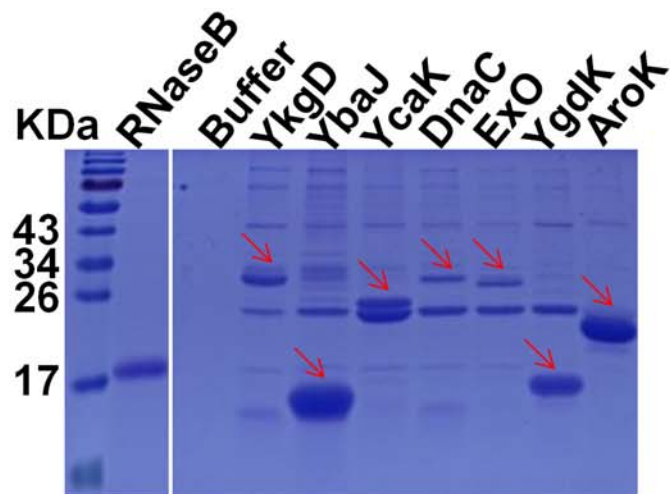

**d.**

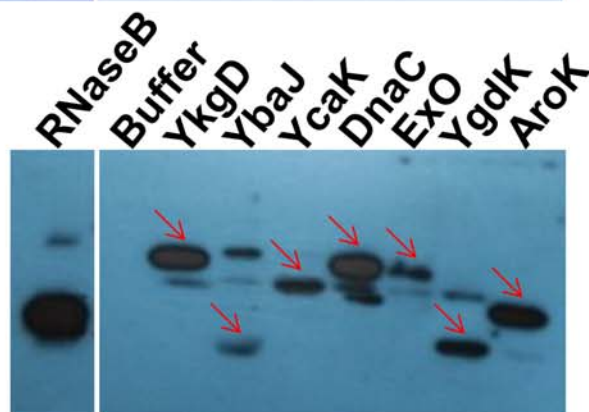

Supplement: Figure S1 — Validation of the 15 successfully purified glycoprotein candidates by lectin blotting. A heavily glycosylated protein RNaseB was included as a positive control. The protein elution buffer used to purify the glycoproteins was used as a blank control. Red arrows indicate the target protein bands on the gel or the membrane. (a, c) Coomassie staining. (b, d) Lectin blotting using a biotinylated WGA followed by HRP-conjugated streptavidin. (PDF) [file pone.0049080.s001.pdf]
