# Supplementary material for: Global Identification of Prokaryotic Glycoproteins Based on an Escherichia coli Proteome Microarray
Source: PLoS One. 2012 Nov 7;7(11):e49080. doi: 10.1371/journal.pone.0049080 (PMC3492326; doi:10.1371/journal.pone.0049080)
Supplement: Table S1 — Biological process: primary metabolic process. (PDF) [file pone.0049080.s002.pdf]

Table S1. Biological process:primary metabolic process.

No.	GO term	Gene_Number	Percentage	Gene_Name
1	nucleobase, nucleoside, nucleotide and nucleic acid metabolic process (GO:0006139)	4	33.33%	deoA,ykgD,ribD,Exo
2	cellular amino acid and derivative metabolic process (GO:0006519)	4	33.33%	Edd,aroK,entC,hisP
3	protein metabolic process (GO:0019538)	2	16.67%	surA,holC
4	lipid metabolic process (GO:0006629)	1	8.33%	ydiD
5	carbohydrate metabolic process (GO:0005975)	1	8.33%	talB
