# Supplementary material for: Global Identification of Prokaryotic Glycoproteins Based on an Escherichia coli Proteome Microarray
Source: PLoS One. 2012 Nov 7;7(11):e49080. doi: 10.1371/journal.pone.0049080 (PMC3492326; doi:10.1371/journal.pone.0049080)
Supplement: Table S2 — Molecular function: catalytic activity. (PDF) [file pone.0049080.s003.pdf]

Table S2. Molecular function:catalytic activity.

No.	GO term	Gene_Number	Percentage	Gene_Name
1	transferase activity (GO:0016740)	4	30.77%	aroK,Exo,deoA,talB
2	hydrolase activity (GO:0016787)	4	30.77%	ribD,hisP,Exo,holC
3	ligase activity (GO:0016874)	1	7.69%	ydiD
4	oxidoreductase activity (GO:0016491)	1	7.69%	maoC
5	deaminase activity (GO:0019239)	1	7.69%	ribD
6	lyase activity (GO:0016829)	1	7.69%	Edd
7	isomerase activity (GO:0016853)	1	7.69%	surA
